# Habitat use and social mixing between groups of resident and augmented bighorn sheep

**DOI:** 10.1038/s41598-019-51370-y

**Published:** 2019-10-18

**Authors:** Rusty W. Robinson, Jericho C. Whiting, Justin M. Shannon, Daniel D. Olson, Jerran T. Flinders, Tom S. Smith, R. Terry Bowyer

**Affiliations:** 1 Department of Plant and Wildlife Sciences, Brigham Young University, Provo, Utah USA; 2 Department of Biology, Brigham Young University-Idaho, Rexburg, Idaho USA; 3 Utah Division of Wildlife Resources, Salt Lake City, Utah USA; 4 Institute of Arctic Biology, University of Alaska Fairbanks, Fairbanks, Alaska USA

**Keywords:** Behavioural ecology, Conservation biology, Restoration ecology

## Abstract

Monitoring dispersal, habitat use, and social mixing of released ungulates is crucial for successful translocation and species conservation. We monitored 127 female bighorn sheep (*Ovis canadensis*) released in three populations from 2000 to 2009 to investigate if augmented bighorns expanded and shifted seasonal ranges, used different habitat compared with resident females, and if animals mixed socially. Augmented bighorns in all populations expanded range use compared with residents by shifting utilization distributions. Size of utilization distributions, however, were smaller for augmented females compared with residents in all areas except one. Overlap of seasonal utilization distributions between augmented and resident bighorns and use of slope and elevation differed across populations. In two populations, differences in size and overlap of seasonal utilization distributions and use of slope and elevation supported the hypothesis that habitat use of bighorns in their source area influenced their habitat use after release. Mixing between resident and augmented adult females occurred on average during only 21% of sightings and was similar across populations. Our results clarify how augmented bighorns mix with resident animals and how habitat use is modified following augmentations. Such information is needed to improve bighorn sheep augmentations and can be applied to augmentations of other ungulates.

## Introduction

Mammals are imperiled worldwide and are of conservation concern^1,2^. Moreover, large-body mammals (e.g., ungulates) are most vulnerable to threats of extinction^3^, and ungulates experience greater threats than their smaller-bodied counterparts^2^. Such threats to extinction have resulted in both historical and contemporary approaches to conserve populations of mammals^4,5^. Indeed, restoration of individuals has been a pivotal approach in attempts to re-establish populations of ungulates^6–8^. Nonetheless, not all attempts at restoring populations of ungulates have been successful^6,9^.

Monitoring dispersal and habitat use of released ungulates is important to understand movement rates, release-site fidelity, feasibility of future releases, and success of reintroductions (i.e., the release of an animal into an area that was once part of its range, but from which it has been extirpated^10^) and translocations (i.e., the movement of organisms from one area to another^10^, often to supplement existing populations)^11–13^. For example, dispersal in North American elk (*Cervus elaphus*) and Alpine ibex (*Capra ibex ibex*) familiarizes these reintroduced animals with their new environment^14–16^. Dispersal also can connect isolated populations^10,11,17^ or reconnect populations in a metapopulation structure^11,18^. With elk, white-tailed deer (*Odocoileus virginianus*), and Chinese water deer (*Hydropotes inermis*) dispersal can be influenced by sex, age, reproductive state, group dynamics, time since release, and distance to anthropogenic structures (e.g., roads, reservoirs, buildings)^13,15,19,20^. These movements, however, can increase mortality via predation and be energetically costly^14,21,22^. Quantifying habitat use of recently released ungulates is critical for conservation and management of these unique animals and their habitat^11,14,23^. Understanding dispersal and habitat use of released animals that are naïve to their surroundings is crucial for successful population establishment^6,24,25^, especially in instances when number of individuals, survival, or reproductive rates are low while animals become accustomed to habitats of their release site^26–28^.

Ungulates released to new areas may adjust dispersal and habitat use based on the presence of and social interaction with resident animals. Gregarious ungulates that are translocated sometimes use different areas because of interference competition with resident animals^29–31^. Conversely, augmented (i.e., the release of individuals into an existing population^10^) animals can slowly assimilate space use with resident animals as occurred for Alpine ibex and Persian fallow deer (*Dama mesopotamica*)^14,29^. Additionally, augmented animals may establish home ranges similar to those of previously released animals^19,29^. Individuals that assimilate with resident animals can increase success of translocations by settling in areas used by resident animals, thereby increasing population size and probability of population persistence^14,32,33^. Behavioral interactions of augmented animals with resident animals, however, are rarely studied and those observations are needed to improve efficacy of translocations^24,34,35^. Indeed, translocations can provide important opportunities for experimentation on the maintenance of traditions of habitat use and the extent to which social mixing influence such use^34,36,37^.

Researchers have reported that only 41% of bighorn sheep (*Ovis canadensis*) translocations were successful^38^. Bighorn sheep reintroductions and translocations have occurred to establish new populations or to ostensibly expand the distributions of existing herds^38–40^. Sedentary, historical populations that receive additional translocated individuals in or near habitat used by animals in those historical populations potentially increase movements and migration of resident and augmented animals^38,39^. Further, small, historical populations of bighorn sheep may have lost all or some of their migratory behaviors and augmentations of animals may help re-establish those traditions^32,40^. This range-use expansion was hypothesized to occur, because these ungulates have open societies and migration patterns, and the use of seasonal ranges are learned and passed through generations^41^. This increase in movements and migration by augmented animals could increase the area used by a population, which could be beneficial for resident animals that underutilize available habitat^32,42^. In one population, however, transplanted bighorns used different ranges and had limited social mixing with resident animals within the first 3 years after release^42^. In that study, the size and extent to which differences in habitat use occurred by season and social mixing between augmented and resident females were not quantified. Reintroductions and translocations of bighorn sheep are likely to proceed at an increasing rate, especially with habitat loss and fragmentation further threatening populations of these ungulates^9,24^. Understanding factors that affect habitat use and social interactions in a new environment is critical for improving augmentations of bighorn sheep, as well as for other social ungulates, such as ibex, chamois (*Rupicapra* spp.), mountain goats (*Oreamnos americanus*), and pronghorns (*Antilocapra americana*)^14^; this is a critical element for their successful conservation.

Habitat use and movement among ungulates in a population increases the probability of individuals encountering conspecifics and transferring information about habitat use^40,41,43^. Much can be learned regarding how augmented animals interact with resident animals, which can improve success of translocations for ungulates worldwide^6,24,44^. In addition, long-term monitoring often is lacking in animal reintroduction and translocation programs^35^, and simultaneous comparisons of augmentation outcomes are rare across multiple populations^35^. We compared habitat use and social mixing of augmented (animals released in 2007) with resident bighorn sheep (animals released from 2000 to 2004) across three study populations, which provided powerful replication for the general application of our findings^24,34,45^. We monitored 127 released adult, female bighorn sheep in three adjacent populations in northern Utah, USA, from 2000 to 2009. We hypothesized that augmented adult female bighorns would use similar habitat and mix socially with resident females^29,37,41^. Specifically, we predicted that augmented bighorns would occupy similar areas (i.e., exhibit comparable size and overlap of 95% seasonal and annual utilizations distributions as determined by the utilization distribution overlap index, because such a response has been documented in other studies^41,46,47^) and use areas of similar slope, elevation, and ruggedness as those used by resident female bighorns. We also predicted that augmented groups of female bighorn sheep would mix with female resident groups, because this response has been documented in previous studies with this species^37,41^. Our results provide important information on how augmented bighorn sheep mix with and use habitat similar to that used by resident animals. Such information is needed to improve our understanding of augmenting bighorn sheep into historical habitat, and can be applied to augmentations of other mountain ungulates.

## Methods

### Study areas

**Figure 1:**
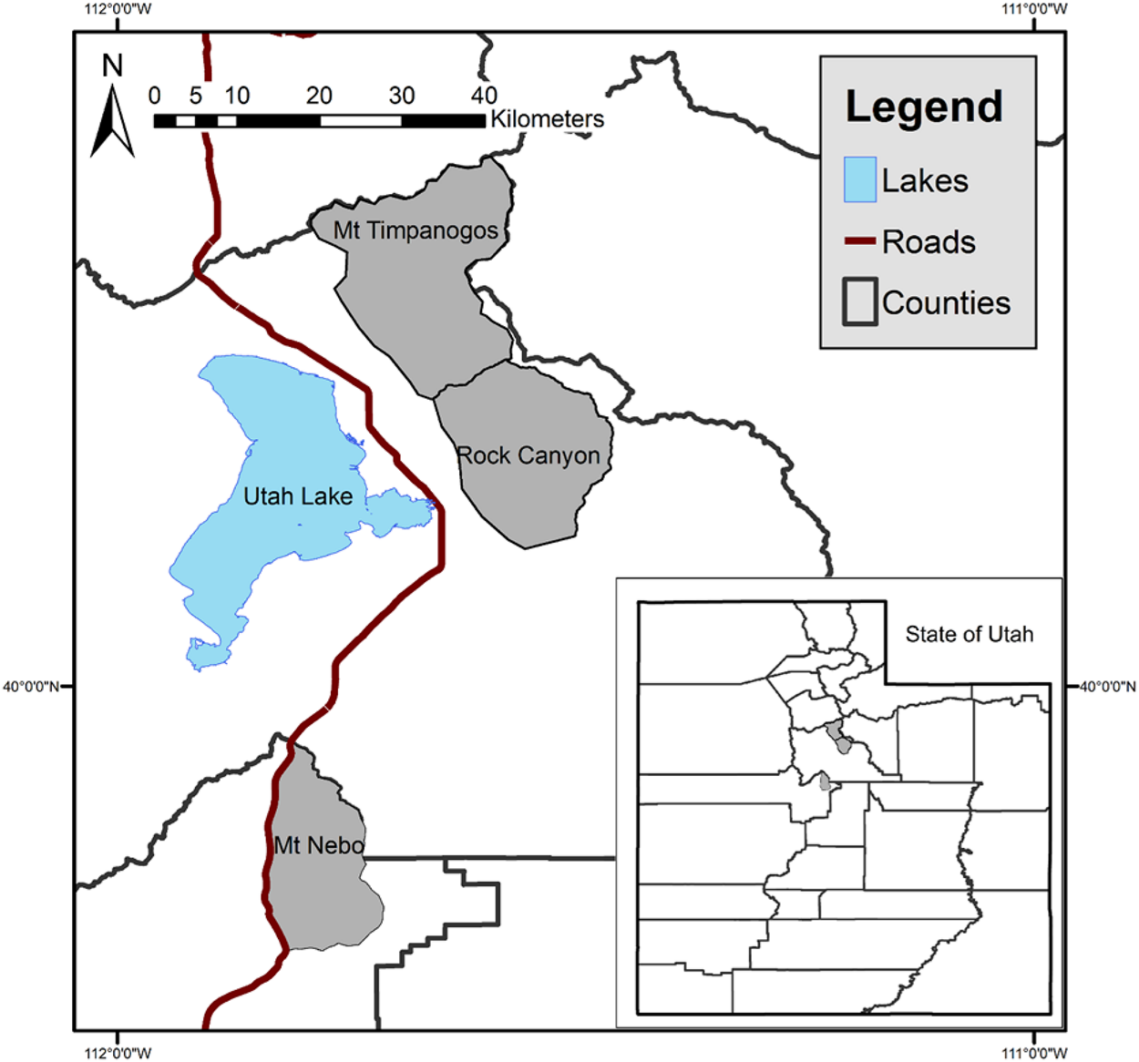
Study areas in which we documented habitat use and social mixing of resident and augmented bighorn sheep in Utah, USA, from 2000 to 2009.

We studied female Rocky Mountain bighorn sheep (*O*. *c*. *canadensis*) that were released to Mount Timpanogos, Rock Canyon, and Mount Nebo in northern Utah, USA (Fig. 1). Elevation in these areas range from 1,388 m to 3,636 m^48^. Mean summer temperature was 19 °C, and average winter temperature was 3 °C^49^. Mean annual rainfall was 51 cm and the average yearly snowfall was 145 cm^50^. Similar topography and flora occurred in all three study areas. Generalized vegetative zones descending in elevation were alpine, conifer, aspen (*Populus tremuloides*), maple (*Acer* spp.), juniper (*Juniperus* spp.), big sagebrush (*Artemisia tridentata* ssp.), forbs, and grasses^48^. Prominent forage species in those areas used by bighorn sheep included bluebunch wheatgrass (*Elymus spicatus*), spike fescue (*Leucopoa kingii*), Sandberg’s bluegrass (*Poa secunda*), shortstem buckwheat (*Eriogonum brevicaule*), and littlecup penstemon (*Penstemon sepalulus*)^51^.

### Field methods

**Figure 2:**
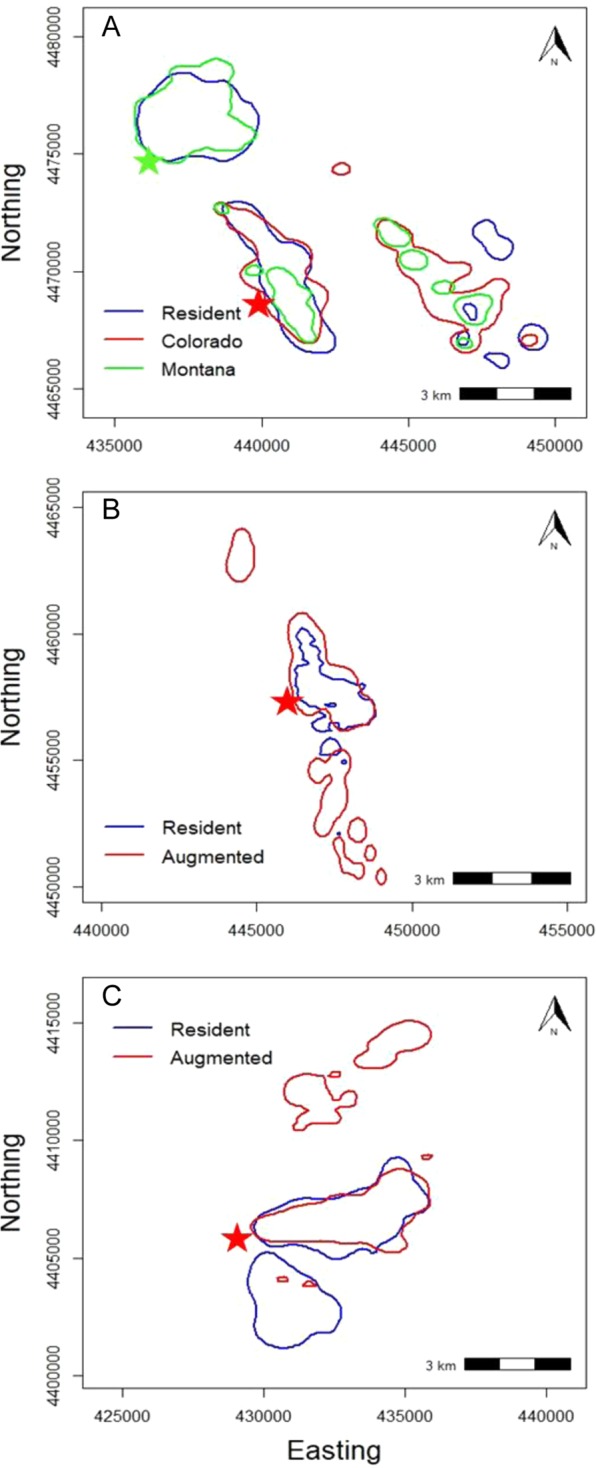
Annual 95% utilization distributions for resident, as well as augmented Montana and Colorado female bighorn sheep on Mount Timpanogos (**A**), Rock Canyon (**B**), and Mount Nebo (**C**) in Utah, USA, from 2000 to 2009. Stars represent the general area where augmented bighorn sheep were released in 2007. Areas on the maps to the east are higher elevations.

All historical populations of bighorn sheep were extirpated from our study areas by the 1930s^52–54^; therefore, from 2000 to 2007, 157 first resident and then augmented bighorn sheep were released to Mount Timpanogos (*n* = 82), Rock Canyon (*n* = 32), and Mount Nebo (*n* = 43) (Table 1). Of those resident and augmented bighorns, Ninety-four females were equipped with VHF radio collars at the time of release (Table 1), and 12 additional females received telemetry collars periodically throughout the study^50^. In releases of resident female bighorn sheep (Mount Timpanogos 2000 to 2002, Rock Canyon 2001, and Mount Nebo 2004; Table 1), adult females in Rock Canyon and on Mount Nebo had colored, numbered ear tags. On Mount Timpanogos, all females had blue ear tags with a unique number from the 2001 release from Alberta, Canada, and females released from Sula, Montana in 2002 had blue ear tags with a unique number. Bighorn sheep augmentations occurred in all areas in 2007, and animals were released in areas near resident bighorn sheep (Table 1, Fig. 2). All our study areas were historical bighorn sheep habitat and identified as suitable release sites by the Utah Division of Wildlife Resources. Wildlife biologists from the Utah Division of Wildlife Resources followed established protocols when handling, translocating, and attaching radio-transmitting collars and ear tags to bighorns^55^. Across all years of our study, mean (±*SD*) population sizes (i.e., all sex and age classes) for these areas were as follows: Mount Timpanogos ($\bar{x}$ = 39, *SD* = 12 animals), Rock Canyon ($\bar{x}$ = 34, *SD* = 16 animals), and Mount Nebo ($\bar{x}$ = 31, *SD* = 15 animals).

**Table 1:** Locations, years of capture, source areas, and demographic information for populations of bighorn sheep released in northern Utah, USA^50^.

Release site and date	Source area	Males	Females	Young	Total	% females collared
***Mount Timpanogos***						
Jan-2000^a^	Rattlesnake Canyon, Utah	6	16	3	25	81
Jan-2001^a^	Hinton, Alberta, Canada	2	8	0	10	100
Feb-2002^a^	Sula, Montana	2	6	1	9	67
Jan-2007	Sula, Montana	0	20	0	20	70
Mar-2007	Alamosa, Colorado	1	17	0	18	100
***Rock Canyon***						
Jan-2001^a^	Hinton, Alberta, Canada	4	15	3	22	67
Jan-2007	Sula and Augusta, Montana	0	10	0	10	60
***Mount Nebo***						
Dec-2004^a^	Augusta, Montana	2	13	3	18	69
Jan-2007	Augusta, Montana	3	22	0	25	59

^a^We considered bighorns from these releases as resident animals.

After initial reintroduction of original bighorn sheep on Mount Timpanogos in 2000, two subsequent releases occurred in 2001 and 2002 totaling 14 females. We considered all bighorn sheep released from 2000 to 2002 on Mount Timpanogos as resident animals (Table 1)^50^, because survival rates for those 14 females released from 2001 to 2002 were low^50^, and similar clumping of ungulates released in subsequent years occurred in another study^14^. Doing such also allowed use to compare resident animals with augmented animals that were released in the same year (2007) in all populations, which helped control for environmental factors that could have influenced movements and space use (e.g., snow depth). We also considered animals released in Rock Canyon (2001) and on Mount Nebo (2004) as resident animals, and those released in 2007 as augmented bighorns (Table 1)^50^.

To differentiate resident from augmented individuals, all bighorns released in all study areas in 2007 were marked with two, colored ear tags. Therefore, after releases in 2007, all augmented females had either a radio-transmitting collar or two ear tags, which facilitated classifying group structure. Consequently, all unmarked females observed after augmentation in 2007, and before April 2009, could be distinguished as either adult residents, lambs (young), or yearlings. Lambs or yearlings observed alone, and therefore indistinguishable from any group, were removed from our analyses (one observation from Rock Canyon, <1%). From April 1 to December 31, 2009, 2-year-old ewes (offspring of augmented females) could not be accurately distinguished from resident uncollared females. In instances when we observed groups of those animals with no collar or ear tag, those locations were removed from our analysis. In total, four locations (1%) were censored from Mount Nebo and 28 (1.7%) were censored from Mount Timpanogos.

We located bighorn sheep with radio collars using radio-telemetry equipment, binoculars, and spotting scopes year-round from 2000 to 2009 on Mount Timpanogos, 2000 to 2008 in Rock Canyon, and 2004 to 2009 on Mount Nebo^49,50,56^. We also observed groups of uncollared bighorns in those same areas and years. We watched groups of bighorns an average of 24 times each month. When we observed a group, we noted sex of individuals and group size and composition^42,51^. We considered animals to be a part of the same group if they were ≤50 m from one another, or if they appeared to be aware of the presence of other sheep and moved as a cohesive unit^51,57^. We only used sightings that contained ≥1 adult, female bighorn sheep for analysis. In all areas, those sightings could include young, yearlings, and males, as long as ≥1 adult female was present in the group.

### Data analyses

To produce 95% utilization distributions and quantify social mixing, we defined bighorn groups as follows: resident groups were ≥1 adult, female bighorn sheep from original releases with any other bighorn sheep (i.e., females, young, yearlings, males) from original releases; augmented groups were ≥1 adult, female bighorn sheep from 2007 augmentations with any other bighorn sheep from 2007 augmentations. Mixed groups consisted of ≥1 adult, resident female with ≥1 adult, augmented female with any other bighorn sheep from any release. When groups of resident and augmented bighorn sheep were mixed, we assigned a location for each group (resident and augmented) for that sighting in our spatial analyses. During our study, two augmented females released in Rock Canyon in 2007 crossed a highway that was considered a barrier to major movements of bighorn sheep and occupied areas on Mount Timpanogos^49^. We censored locations from those females in our analyses, because their inclusion artificially overestimated 95% utilization distributions for augmented bighorns in Rock Canyon.

We calculated size (km^2^) of seasonal and annual 95% kernel utilization distributions^47,58,59^ by study area and group (resident or augmented) using direct plug-in methodology with select bandwidth^60,61^ in R^62^, and only produced utilization distributions for areas or seasons with ≥18 locations. We determined seasons by plotting precipitation against temperature, which had been done previously for these areas^48^. The following four seasons were evident for our study areas: spring (March–May), summer (June–September), autumn (October), and winter (November–February)^48^. Because October was a transitional month^48^, and sample sizes were <18 for all study areas during that month, we did not produce seasonal utilization distributions for autumn. Autumn locations, however, were included in annual utilization distributions.

We quantified the degree of overlap in seasonal and annual utilization distributions by study area and group (resident or augmented) using the utilization distribution overlap index (UDOI)^46,47,63^. UDOI is a three dimensional index that considers the volumetric overlap between utilization distributions. UDOI values typically range from 0 to 1, with 0 indicating no overlap of distributions and 1 indicating 100% overlap of uniform distributions. UDOI can be >1 when utilization distributions are non-uniformly distributed and have a high degree of overlap^46^. We used the KernSmooth package^60^ for kernel smoothing and density estimation and the adehabitat package^63,64^ for utilization distribution analyses and mapping in R^62^. We also compared utilization distributions for resident animals before and after augmentation to assess any changes in distributions for resident animals after augmentations. We used the 2-sample *Z*-test for proportions^65^, which allowed sampling with replacement, to investigate if the proportion of mixed female groups was different among populations. We also used a Bonferroni correction to adjust alpha for our comparisons; that adjusted alpha was 0.02.

We compared use of slope, elevation, and ruggedness for each observation between groups of resident and augmented bighorn sheep^42,57,66,67^ using data from a United States Geological Survey 30-m digital elevation model. Slope was calculated with the slope tool in the Spatial Analyst Tools extension in ArcGIS. We quantified elevation at the site of each location by extracting that value from the digital elevation model. Ruggedness was calculated using the Vector Ruggedness Measure Tool in the Terrain Tools extension in ArcGIS^67^. That tool measures terrain ruggedness as the variation in three-dimensional orientation of grid cells within a neighborhood. Vector Ruggedness Measure values can range from 0 (no terrain variation) to 1 (complete terrain variation). Typical values for natural terrains range between 0 and about 0.4^67^. We used a 95% confidence interval of the difference of means to test for differences in bighorn use of slope, elevation, and ruggedness between groups within study areas^68^.

## Results

### Mount Timpanogos

We used 1,613 sightings to produce seasonal and annual 95% utilization distributions for groups of female bighorn sheep on Mount Timpanogos (Table 2). Augmented female bighorn sheep had smaller annual utilization distributions compared with that of residents (Table 2). On average, size of 95% seasonal utilization distributions for resident bighorn sheep were 2 times larger than estimates of utilization distributions for augmented females (*SD* = 0.47, range = 1.5 to 2.7 times larger, Table 2), with the largest area used by all bighorns in summer, and the smallest area used by all females in spring and winter. Augmented female bighorn sheep from Colorado had smaller utilization distributions, except in summer, than utilization distributions of resident and Montana bighorns (Table 2).

**Table 2:** Study areas, number of observations, and size (km^2^) of annual and seasonal 95% utilization distributions for resident and augmented female bighorn sheep in three populations in northern Utah, USA, from 2000 to 2009.

Study Areas	*n*	Annual	Winter	Spring	Summer
***Mount Timpanogos***					
Resident	922	19.5	13.7	14.1	17.2
Aug. Montana	364	14.2	7.1	7.3	11.0
Aug. Colorado	327	13.1	6.2	5.2	11.8
***Rock Canyon***					
Resident	579	4.2	2.9	2.3	4.2
Augmented	129	6.6	4.2	4.7	5.6
***Mount Nebo***					
Resident	146	12.3	6.4	8.1	8.9
Augmented	256	10.2	4.2	2.1	7.6

Annual 95% utilization distributions for resident bighorn sheep before compared with after augmentation exhibited a high degree of overlap on Mount Timpanogos (UDOI = 1.09), indicating little change in range use of resident animals before compared with after augmentations. After augmentation of bighorn sheep in 2007, overlap of seasonal and annual 95% utilization distributions were substantial between augmented females from Montana and residents (Table 3, Fig. 2). Overlap of annual and seasonal 95% utilization distributions were markedly lower, however, for augmented females from Colorado compared with resident females, with the lowest seasonal overlap occurring in winter and summer (Table 3, Fig. 2). On average, augmented Montana bighorns used areas 68 m higher in elevation compared with resident animals; augmented Colorado bighorns on average used areas 333 m higher in elevation than resident animals (Table 4). No difference occurred with use of slope or ruggedness between resident and augmented animals on Mount Timpanogos (Table 4). Overall, augmentations in this area expanded range use by 5.2 km^2^ (Fig. 2).

**Table 3:** Amount of overlap of seasonal and annual 95% utilization distributions using the utilization distribution overlap index between resident and augmented female bighorn sheep in three study areas in Utah, USA, from 2000 to 2009.

	Resident											
	Mount Timpanogos				Rock Canyon				Mount Nebo			
	Winter	Spring	Summer	Annual	Winter	Spring	Summer	Annual	Winter	Spring	Summer	Annual
**Augmented**												
Montana	1.23	1.11	1.08	1.23	0.83	0.43	0.80	0.80	0.55	0.38	0.56	0.63
Colorado	0.19	0.38	0.22	0.32	—	—	—	—	—	—	—	—

**Table 4:** Abiotic factors used to compare differences in habitat use for groups of resident and augmented bighorn sheep on Mount Timpanogos (MT), Rock Canyon (RC), and Mount Nebo (NB) from 2000 to 2009, Utah, USA.

Abiotic Factor	Group	*n*	$\bar{{\boldsymbol{x}}}$	*SE*	LCL	UCL
Slope	MT Resident	922	69.7	29.9	−4.028	2.63
	MT Colorado	327	69	29.9		
	MT Resident	922	69.7	29.9	−0.97	5.43
	MT Montana	364	71.9	29.9		
	RC Resident	579	81.8	29.9	−14.9	−4.83
	RC Augmented	129	72	30		
	NB Resident	146	67.4	2.73	−2.87	7.85
	NB Augmented	256	69.9	21.17		
Elevation	MT Resident	922	1989	425.4	286.5	381
	MT Colorado	327	2322	425.8		
	MT Resident	922	1989	425.4	22.4	113.3
	MT Montana	364	2057	425.8		
	RC Resident	579	1907	425.5	−107.1	35.9
	RC Augmented	129	1871	426.6		
	NB Resident	146	2504	38.8	184.7	335.5
	NB Augmented	256	2764	303.7		
Ruggedness	MT Resident	922	0.016	0.02	−0.0034	0.0011
	MT Colorado	327	0.015	0.02		
	MT Resident	922	0.016	0.02	−0.0024	0.0019
	MT Montana	364	0.016	0.02		
	RC Resident	579	0.016	0.02	−0.0059	0.0009
	RC Augmented	129	0.014	0.02		
	NB Resident	146	0.015	0.0018	−0.0052	0.002
	NB Augmented	256	0.013	0.014		

We depict sample size, means, standard errors (SE), as well as lower confidence limits (LCL) and upper confidence limits (UCL) for the 95% confidence intervals of the difference in means.

### Rock Canyon

We used 708 sightings to produce seasonal and annual 95% utilization distributions for groups of female bighorn sheep in Rock Canyon. Augmented female bighorn sheep had a larger annual utilization distribution compared with that of residents (Table 2). On average, size of 95% seasonal utilization distributions for resident bighorn sheep were 1.6 times smaller than estimates for augmented females (*SD* = 0.88, range = 1.3 to 2.1 times smaller, Table 2), with the largest area used by all females in summer, and the smallest area used by resident females in spring.

Annual 95% utilization distributions for resident bighorn sheep before compared with after augmentation exhibited a high degree of overlap in Rock Canyon (UDOI = 1.00), indicating little change in seasonal range use of resident animals before compared with after augmentations. After augmentation of bighorn sheep in 2007, overlap of seasonal and annual 95% utilization distributions occurred between augmented and resident females, with the least overlap occurring in spring (Table 3, Fig. 2). A difference in use of slope occurred, with resident animals using areas of steeper slope than augmented females (Table 4). No difference occurred with use of elevation and ruggedness between resident and augmented females in Rock Canyon (Table 4). Augmentation expanded range use by 3.9 km^2^ (Fig. 2).

### Mount Nebo

We used 402 sightings to produce seasonal and annual 95% utilization distributions for groups of female bighorn sheep on Mount Nebo (Table 2). Augmented female bighorn sheep had a smaller annual utilization distribution compared with that of residents (Table 2). On average, size of 95% seasonal utilization distributions for resident bighorn sheep were 2.2 times larger than estimates for augmented females (*SD* = 1.50, range = 1.2 to 3.9 times larger, Table 2), with the largest area used by all females in summer, and the smallest area used by augmented females in spring.

Annual 95% utilization distributions for resident bighorn sheep before compared with after augmentation exhibited low overlap on Mount Nebo (UDOI = 0.44), indicating that resident females changed seasonal range use after augmentation. That low overlap was likely because of 7 of 8 collared resident females dying of disease within 11 months after augmentation. After augmentation of bighorn sheep in 2007, overlap of seasonal and annual 95% utilization distributions between augmented and resident females was minimal, with the least overlap occurring in spring (Table 3, Fig. 2). On average, augmented females used areas 260 m higher in elevation compared with resident animals (Table 4). No difference occurred with use of slope or ruggedness between resident and augmented females on Mount Nebo (Table 4). Augmented females expanded range use by 6.5 km^2^ (Fig. 2).

### Social interactions

**Figure 3:**
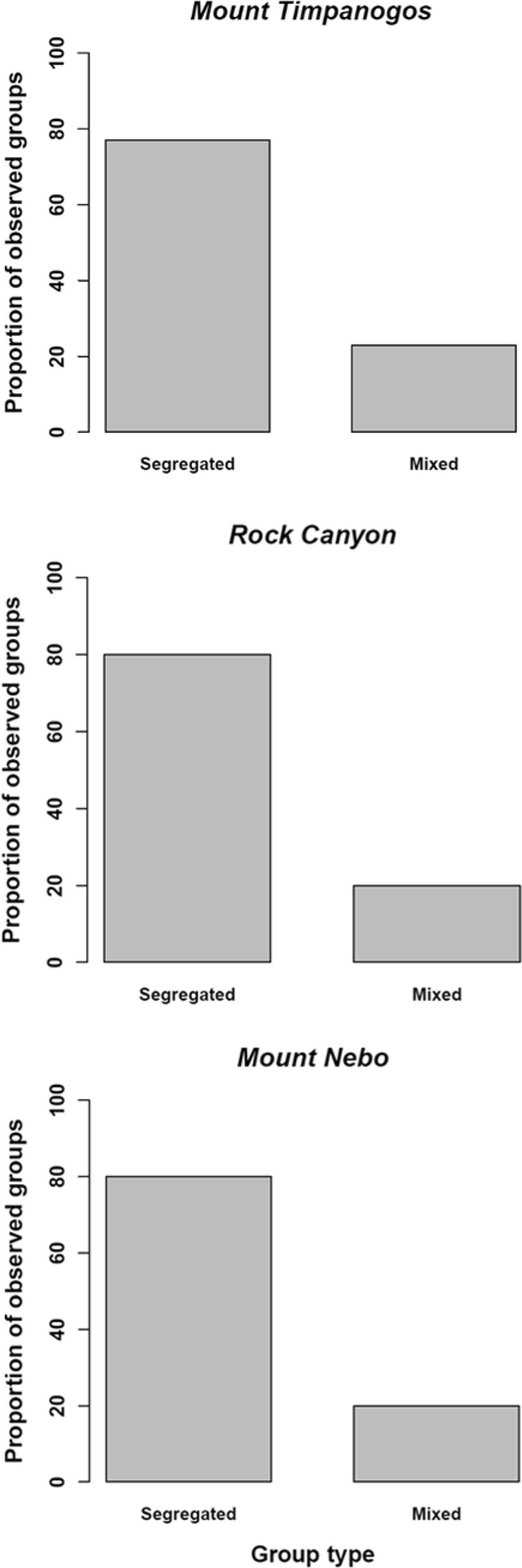
Proportion of segregated and mixed groups of resident and augmented bighorn sheep in three study areas in Utah, USA, from 2007 to 2009.

From 2007 to 2009, we observed 1,467 groups of bighorn sheep (Mount Timpanogos = 880 groups, Rock Canyon = 273 groups, and Mount Nebo = 314 groups). The proportion of mixed female groups did not differ among populations (Mount Timpanogos and Rock Canyon, *Z* = 0.98, *p* = 0.32; Mount Timpanogos and Mount Nebo, *Z* = 0.82, *p* = 0.41; Rock Canyon and Mount Nebo, *Z* = −0.18, *p* = 0.86; Fig. 3). On Mount Timpanogos when groups were mixed, resident bighorns mixed with Montana bighorns 64% of sightings; while resident bighorns mixed with Colorado animals 6% of sightings. Montana and Colorado bighorns mixed 22% of sightings, and all three groups mixed 8% of the sightings.

## Discussion

### Similarities among augmentations

Augmented bighorn sheep in all populations expanded range use by shifting utilization distributions after augmentation regardless of how long resident bighorns had been in the area. Similar range expansion has been documented in other populations^42,69^. That shifting of utilization distributions of augmented adult females is important to wildlife conservationists who want to expand areas used by existing populations, which can help with connectivity and population persistence^39,70,71^. Our results indicate that augmenting populations can increase habitat use by bighorn sheep. A unique aspect of our study was quantifying range expansion by seasons. That understanding is important for bighorn sheep population persistence^39,71,72^. Across all populations, the seasons with the smallest utilization distributions by augmented bighorns were winter and spring. Those outcomes likely occurred because winter range was limited, resulting in animals moving to lower elevations during winter, and after winter subsequently moving to smaller areas for birthing. Additionally, the season with the largest size of utilization distributions for augmented animals was summer. Therefore, releasing bighorn sheep in areas with connectivity to large areas of underutilized summer habitat has the potential to expand range use.

Augmented animals can learn information by socializing with others^25,29,73^, and understanding social relations by animals can improve wildlife conservation^40,74^. Comparatively little work, however, has been done on the behavior of released animals^24,35,36^, and is needed to improve translocations^34^. Indeed, animal augmentations provide unique opportunities for understanding social interactions^34^. In our study, mixing between resident and augmented adult females occurred infrequently. Intriguingly, the frequency of group mixing across populations was similar, indicating a consistency in the lack of social mixing of augmented bighorn sheep with residents on our study sites. Low levels of social interaction between females in an historical population and augmented females have been documented anecdotally in one population of bighorn sheep^69^. Female bighorn sheep from historical populations may recognize individuals from their own group and may not readily join other groups even if their range use overlaps^75^. Additionally, low social interactions occurred between juvenile (<3 years old) translocated bighorn sheep and bighorns from an historical population in nursery groups in summer in one population^37^. Those low social interactions between juvenile augmented bighorn sheep and animals from that historical population affected time spent foraging and body mass of augmented individuals, as well as possibly translocation success^37^. Future research should verify if group mixing between resident and augmented adult females increases with a longer time (>2 years) after release.

#### Augmented Montana bighorns on Mount Timpanogos and Mount Nebo

Resident female bighorn sheep used seasonal areas that were on average almost twice as large as seasonal estimates for augmented females from Montana. That difference in use of larger seasonal areas may have been because resident bighorns had been in those areas for 7 (Mount Timpanogos) and 2 years (Mount Nebo) prior to release of augmented bighorn sheep, and had sufficient time to become more familiar with their environment and thereby use more habitat^25^. Further, overlap of utilization distributions for resident females on Mount Timpanogos did not change after augmentations, indicating that augmentations had little effect on utilization distributions of resident animals. Overlap of seasonal utilization distributions were substantial, however, between resident and augmented bighorn sheep on Mount Timpanogos, indicating that augmented bighorns used similar, smaller areas than those used by resident bighorn sheep. Additionally, social mixing was highest between those two groups, which was most likely driven by the significant overlap in utilization distributions between resident and augmented, Montana bighorn sheep on Mount Timpanogos. Similar to Colorado bighorns on Mount Timpanogos, augmented bighorns on Mount Nebo used areas of higher elevation, and seasonal overlap in habitat use was low, indicating that augmented animals expanded range use to areas of higher elevation, especially in spring.

### Differences among augmentations

#### Augmented Colorado bighorns on Mount Timpanogos

As with augmented bighorns from Montana, resident female bighorn sheep used seasonal areas that were on average almost twice as large as seasonal estimates for augmented females from Colorado. Again, that difference in use of large seasonal areas by resident animals likely was because resident bighorns had been in that area for 7 years prior to release of augmented animals^25^. Additionally, overlap of seasonal utilization distributions was low between resident and Colorado bighorn sheep. That lack of overlap was caused by augmented bighorns using areas of much higher elevation in summer and moving to different areas than resident bighorns in winter, which also resulted in the lowest social mixing between those groups. Indeed, bighorn sheep from Colorado came from a source herd where many of those animals use year-round habitat at high elevations (>3,000 m).

Addressing *a priori* questions in reintroduction biology allows biologists to use scientific evidence to apply best management practices in the field^26,35^. Biologist from the Utah Division of Wildlife Resources released bighorns from Colorado on Mount Timpanogos to improve the use of high-elevation habitat. That release did expand range use to areas of high elevation, and our data support the hypothesis that habitat use of a source population can influence habitat use by animals after release^30^. Indeed, Mount Timpanogos offered a unique opportunity to compare augmentations from different source habitats; such replication often is lacking in studies of reintroduction biology^30,35^.

#### Rock canyon

Unlike resident female bighorn sheep on Mount Timpanogos and Mount Nebo, resident females in Rock Canyon used seasonal areas that were on average 1.6 times smaller than areas used by augmented female bighorns. That reduction in area used by resident females likely occurred because those animals moved to small rocky areas of steeper slope to give birth that overlapped little with augmented animals, similar to what occurred in other areas. Resident bighorn sheep also used small winter ranges compared with augmented animals. In addition, birthing and wintering areas for resident animals were near the release site, indicating a lack of dispersal. Also, overlap of utilization distributions for resident females did not change after augmentations, again indicating that augmentations had little effect on utilization distributions of resident animals. Resident bighorn sheep in Rock Canyon came from a mine site near Cadomin, Canada. Bighorns in that area have small seasonal ranges on the reclaimed mine site; therefore, we hypothesize that limited movements of resident animals in Rock Canyon at their capture site influenced habitat use by those animals after release. Our results from resident females in Rock Canyon add support for the hypothesis that habitat use of a source population can influence habitat use by animals after release^30,56^. Additional research should test if habitat use by augmented animals and resident animals becomes similar with longer time after release.

### Density-dependent factors

Population size was low and similar in our three study areas. Additionally, female bighorns in our study populations experienced high pregnancy rates indicating that those populations were not likely influenced by density-dependent factors^48–50^. As documented in all of our study populations, augmented animals expanded range use by shifting utilization distributions after augmentation. That expansion could have occurred because resident bighorn sheep were at low densities and had not occupied all available habitat in each study area. Testing ideas about density dependence while monitoring released animals is needed^34^. For example, small populations, or low densities, may be less attractive than larger ones for released animals^36^, thus potentially influencing habitat use and social mixing. We hypothesize that in areas with resident bighorn sheep at higher population densities than ours, that range expansion by augmented animals may not be as pronounced as what we observed. Further, we predict that social interactions would occur more often when augmented bighorn sheep are released into areas with resident bighorn sheep at high population densities^34^. Future research can test those ideas.

### Social interactions and learning

Habitat use and social mixing of bighorn sheep can be influenced by learning that is shaped by predator avoidance and feeding efficiency^41,76,77^. This learning can influence habitat use and social mixing of younger animals especially, as they interact with and follow older individuals^41,78,79^, or as they form groups to avoid predation^41,76,77^. Furthermore, movement to and use of the seasonal distribution of high-quality forage by translocated bighorn sheep appears to be culturally transmitted across decades, with knowledge acquired of such areas of forage increasing over time since release^40^. Bighorns we studied that were considered resident (animals released from 2000 to 2004) may have still been familiarizing themselves with habitats in our study areas^40^; therefore, we caution about comparing our results to those of translocations of bighorn sheep into established historical populations. In our study areas, we hypothesize that habitat use and social mixing between resident and augmented adult females will increase as migration traditions are inherited over generations^40^.

### Management implications

Populations of bighorn sheep have declined substantially since the late 1800s^41,52,80^. Augmentations of bighorn sheep are often used to re-establish populations into historical habitat and to supplement declining herds^38,39,42^. Despite those efforts, success rate of translocated populations of bighorns is low^38,42,80^; indeed, suitable habitat and connectivity between augmented and resident animals is crucial for successful bighorn sheep augmentations^32^. We were able to quantify habitat use and social mixing of augmented and resident bighorn sheep across three study populations, that replication was crucial for the generalization of findings^24,35,45^. We documented range expansion by all augmented bighorn sheep as they shifted utilization distributions. Size of utilization distributions, however, were smaller for augmented females compared with residents in two of three areas. Differences in size and overlap of seasonal utilization distributions and use of slope and elevation in two populations supported the hypothesis of habitat use of animals in their source areas transferring to release areas after augmentation^30^. Finally, mixing between resident and augmented adult female bighorn sheep occurred infrequently, and was similar across populations. Our results provide important information on how augmented bighorn sheep mix with resident animals and how use of habitat changes after releases. Such information is needed to improve augmentations of bighorn sheep, and can be applied to releases of other open-habitat social ungulates as well.
